# A Prize of $300 for Essay or Treatise on Teaching and Training Deaf Children

**Published:** 1917-02

**Authors:** 


### A Prize of $300 for Essay or Treatise on Teaching and Training Deaf Children.

The American Association to Promote the Teaching of Speech to the Deaf is authorized to pay $300 of the income received from the Alexander Graham Bell Grosvenor Memorial Fund for the essay, treatise, or other form of composition complying with the following conditions that most clearly details how a mother can best teach and train her deaf child in the home from infancy to school age:

Each essay submitted must (1) be delivered at the Volta Bureau, by prepaid express or mail, before 12 o'clock noon of November 1, 1917; (2) must be typewritten in the English language; (3) must contain at least 20,000 words, as it is doubtful if the necessary instructions and suggestions can be properly presented with a less number; illustrations may be used if the author prefers; (4) must bear a distinguishing mark or pseudonym, but nothing to tell who the author is or where residing; (5) must not be folded or rolled, but placed in a large, plain envelope bearing only the title of the essay and the distinguishing mark of the author; (6) must be accompanied by a small envelope bearing the title and distinguishing mark on the outside and containing the name and address of the author in a signed statement that the essay is entirely the writer's own production; (7) must be wrapped and addressed to The Judges for the Alexander Graham Bell Grosvenor Memorial Fund Prize, Volta Bureau, 1601 Thirty-fifth Street, N. W., Washington, D. C.

The judges elected to pass upon the merits of the offerings are:

Mr. and Mrs. Edmund Lyon, Rochester, N. Y.; Dr. and Mrs. A. L. E. Crouter, Mt. Airy, Philadelphia, Pa.; Mr. and Mrs. Gilbert H. Grosvenor, Washington, D. C.

These judges will render a report to the Directors of the Association, who reserve the right to withhold the prize should the judges report that none of the compositions possess sufficient merit to warrant making an award.

The composition awarded the prize becomes the property of the American Association to Promote the Teaching of Speech to the Deaf, to be published where and under such conditions as the directors may determine.

If further details are required, do not address the judges, but write to the Superintendent of the Volta Bureau, 1601 Thirty-fifth Street, N. W., Washington, D. C.

---

Dr. W. G. Jameson, for so many years the Chief Surgeon of the I. & G. N. Railway, died at his home in Palestine, February 13, 1917. Dr. Jameson was well known and much loved. He will be greatly missed in medical circles.

